# Life unlocking card game in death and dying classroom for medical students

**DOI:** 10.15694/mep.2018.0000181.1

**Published:** 2018-08-22

**Authors:** Tharin Phenwan, Tanida Apichanakulchai, Ekkapop Sittiwantana

**Affiliations:** 1 Walailak University School of Medicine; 2 Buddhika Foundation

**Keywords:** Advance Care Planning, Gamification, Thanatology, Qualitative Research, Palliative Care, Pre-clinic students, Behavoiural sciences

## Abstract

This article was migrated. The article was marked as recommended.

**Introduction:** Advance Care Planning (ACP) is a part of comprehensive palliative care but there are challeges for its implementation. In Thailand, undergraduate medical curriculum also has not implemented palliative care and ACP as a core teaching topic for the medical students yet. Life Unlocking Card Game is an end-of-life conversation card game that aims to bridge this gap.

**Objective:** To assess second year medical students’ attitude of death by using Life Unlocking Card Game and its effectiveness to teach about death and dying.

**Methods:** Non-equivalent quasi-experimental design with convenience sampling method. All (48) of second year medical students participated in an end-of-life conversation game (8 games in total with one facilitator within each group). After that, each group formed an after-game focus group interview. Seven students also joined individual semi-structured interviews. We used content analysis approach along with investigator triangulation and methodological triangulation methods for the qualitative analysis.

**Results:** Participants (n = 48) were second-year preclinical medical students. 26 of them were male (54.2%), 22 were female (45.8%), with the mean age of 20 years (SD 0.6). Five primary themes regarding the card game emerged: 1) Safe space to disclose personal issues 2) Seeing the world through different views 3) Surprise elements 4) Death distant closure 5) Changed behaviour.

**Conclusions:** Life Unlocking Card Game proves to be an effective tool to teach death and dying issues and also ACP in second year medical students. Further study in clinical year students or postgraduate students are recommended.

## Introduction

Advance Care Planning (ACP) is one of the essential elements to deliver comprehensive palliative care, an approach that aims to improve patient and family’s quality of life in incurable diseases WHO | WHO Definition of Palliative Care [Internet]. Who.int. 2016 [cited 30 November 2016]. Available from: http://www.who.int/cancer/palliative/definition/en/. The purpose of ACP is to reach a mutual agreement between the health care team, patients, and family if the patient become incapacitated ( Messinger-Rapport, Baum and Smith, 2009). Apart from the medical treatment options, an ACP also helps an individual reflect about their psychosocial issues or other unfinished businesses as well, emphasising the importance of ACP ( Messinger-Rapport, Baum and Smith, 2009). However, making an ACP is still a big challenge in most countries ( Sudore and Fried, 2010).

In Thailand, there has been National Health Act legislation since 2007 Living Will in Thailand - Isaan Lawyers - Attorneys in Thailand. 2016 [cited 24 February 2017]. Available from: http://www.isaanlawyers.com/living-will-thailand/. This Act enables any Thai citizen to write a living will and clarify their needs. However, living wills are still not widely recognized. The prevalence of living wills uptake in Thailand is still unknown. Social context that perceived death as a sensitive subject is another challenge that make it harder to talk about ACP ( Walter, 2003; Meeuwesen, van den Brink-Muinen and Hofstede, 2009; S. Stonington, 2011; S. D. Stonington, 2012), reflecting Hofstedt’s work that describe Thailand as a country with high certainty avoidance dimension and prefers not to confront any ambiguity Hofstede GH, Hofstede GJ, Minkov M. Cultures and organizations : software of the mind : intercultural cooperation and its importance for survival. 3rd ed. New York: McGraw-Hill; 2010. xiv, 561 p. p. Another contributing factor is physicians’ lack the knowledge to initiate a proper conversation about ACP(Mack *et al.*, 2010; Mack *et al.*, 2012) even though most patients and their relatives want to talk about it ( Astrow *et al.*, 2007; Best, Butow and Olver, 2014; Rodin *et al.*, 2015; Brown *et al.*, 2016). For the undergraduate medical curriculum, Suvarnabhumi et al. performed a survey in 2013 and found that only 61% of last year medical students stated that they were taught in ACP with only 33.3% of this group felt that they felt competent to initiate ACP by themselves ( Suvarnabhumi *et al.*, 2013).

To reduce those gaps, there are several recommendation and interventions; e.g. conversation card game ( Van Scoy *et al.*, 2016a; Van Scoy *et al.*, 2016b; Van Scoy *et al.*, 2016c). Several studies show that by engaging in an end of life conversation card game, participants find it safer and easier to talk about their death and ACP(Van Scoy *et al.*, 2016a) while some also completed their advance directive afterward ( Van Scoy *et al.*, 2016b; Van Scoy *et al.*, 2016c). The Life Unlocking Card Game is a conversation card game that aims to raise ACP awareness and engage in talking about death and dying in a safe environment. This pilot study aims to assess undergraduate medical students’ attitude of Life Unlocking Card Game and its effectiveness to teach about death and dying.

## Life Unlocking Card Game

**Figure 1.**
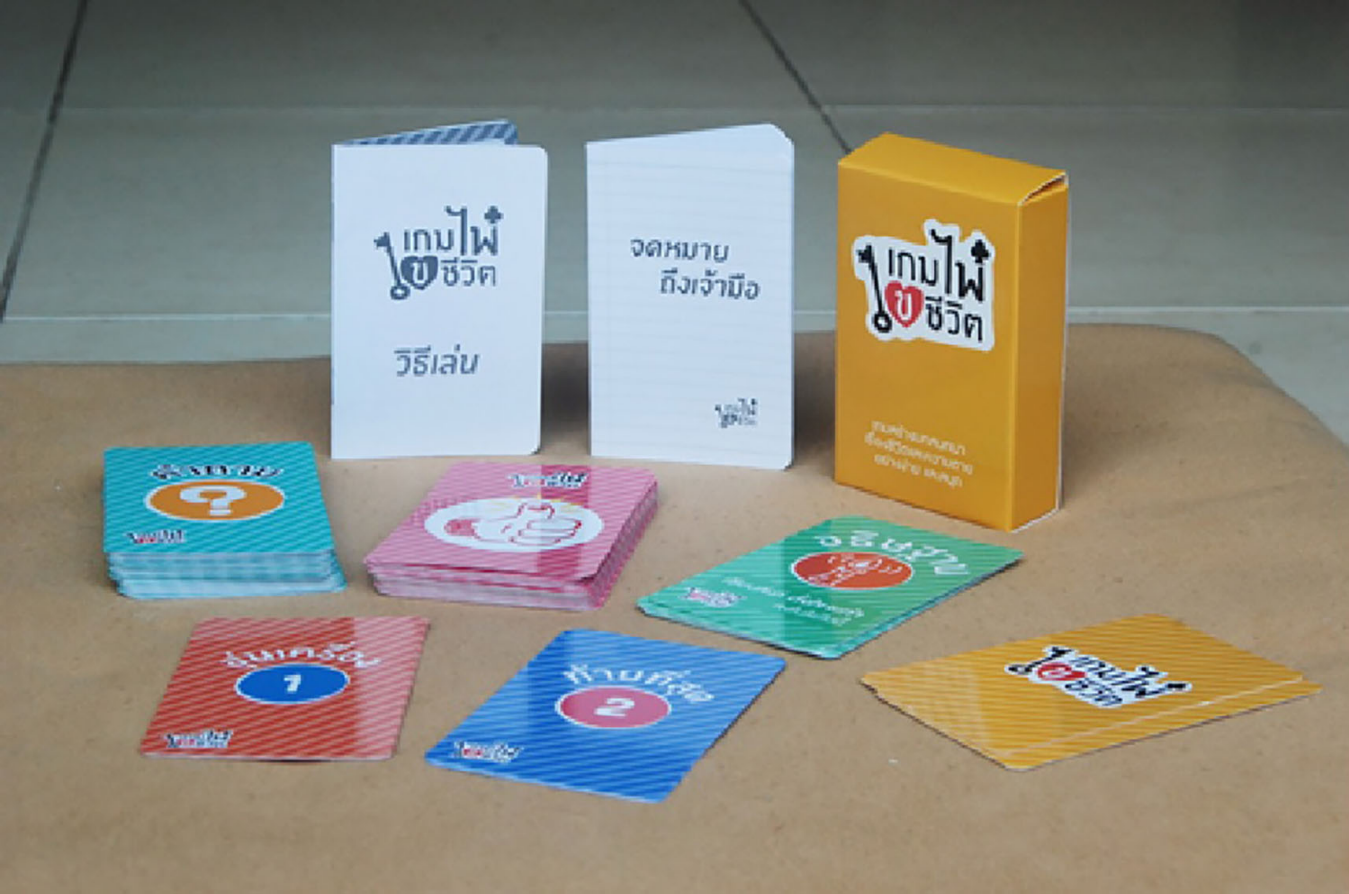


*Gem Pai Khai Cheevitr* (Life Unlocking Card Game) consisted of 45 questions grouped into three subcategories; 1) Emotional respite, 2) Death and Dying Issues and 3) ACP. The card game was conceived with the objective to use the gamification element to talk about hard and sensitive topics such as ACP and death and dying issue เกมไพ่ ไขชีวิต (no date) เกมไพ่ ไขชีวิต | โครงการเผชิญความตายอย่างสงบ. Available at: http://boonbudnet.com/sunset/node/403 (Accessed: December 8, 2017). It aims to raise the awareness of death and dying issues and the importance of ACP. Each session consisted of 4-10 players with one facilitator to guide the group ( Figure 1).

### Reliability, Validity and, Generalisability ( Leung, 2015)

After the conception, the first draft was coined and tested with 20 team members in the Buddhika foundation. After several revisions, the second draft was retested with 30 more people. After that, we conducted the card games for 12 sessions with participants from various background during late 2014 to early 2015; volunteers, healthcare providers who were actively involved in the palliative care service, and those who are interested in the game. For the healthcare professionals, we purposively selected those who were actively working in palliative care in a different setting, policymakers, those who worked in medical schools or teaching hospitals, those who worked in the community, to provide data-rich information and feedback for the game. As for the other participants, we used advertisement through social media and newsletters to recruit anyone who was older than 18 years old and was interested in playing the game. The questions were revised from the feedback to make them more practical and concise ( Appendix I). The full report of the feasibility study and analysis of the pilot card game is currently under review elsewhere and is not available yet.

### How to play

The session started with two warm-up questions to establish rapport between the players; 1) What are your worries about playing this game? 2) What is your expectation from playing this game? After that, players then shuffled the deck, drew the top card, and read the question out loud. Once a question card was drawn, each of them would think or write their answers on a piece of paper. They then took turns explaining and discussing their answers within the group. Participants could give “like” card to players that made insightful comments or to show their support and gratitude to that player. After everybody took turns in answering the question, another player drew a card, and the cycle went on. The session lasted for 90 to 120 minutes. The game concluded with two final questions; 1) What did you learn from this game? 2) What would you do after this game?

## Methodology

Non-equivalent quasi-experimental design with convenience sampling method. We used O’Brien’s Standard for Reporting Qualitative Research as a framework to report our findings ( O’Brien *et al.*, 2014). All (48) of Walailak University School of Medicine second-year students in the academic year 2017 joined the study. They were divided into eight groups with six students and one facilitator within each group. Primary researcher (TP) initiated the session and introduced the students to the trained facilitator team (TA, and six other facilitators). We mixed the students between those who have recently lost someone close to them within one-year time and those who have not. After that, the conversation card game started.

### Data collection and analysis

After the participants wrote their final answers, they joined post-game focus group interviews to give feedback regarding 1) their experience and attitude towards the card game 2) its practicability in understanding the concepts of death and ACP 3) their opinion of this teaching method. Seven students also joined the semi-structured interviews afterwards. All interviews were audio recorded and transcribed verbatim. TP wrote field notes and audit trails immediately after each interview session. Data were gathered until we reached saturation, the point where no new concepts emerged. Demographic data were reported as frequencies and percentages for categorical variables with means and standard deviations (SDs) for continuous variables. For the qualitative analysis, we used investigator triangulation and methodological triangulation methods. Each researcher created individual codes and discussed any discrepancies before making the final codes using content analysis approach. Data from participants also came from multiple sources; focus group interview, semi-structured interview, students’ feedback, and their last wish card. Atlas.Ti 8.0 was used for the coding process.

After three months, TP made follow-up phone calls and asked if the students changed their behaviour according to what they wrote in their last wish card and assessed students’ behaviour using Prochaska’s transtheoretical model (TTM). TTM is a model presumed that behavioural change can occur in 5 stages of change overtime and that proper intervention is needed at each stage to make participants change their behaviour ( Prochaska and Velicer, 1997). For this work’s context, we focused on students’ behaviour and attitude regarding death and ACP from the interviews after the game and their last wish cards. 5 stages of change are: pre-contemplation (death is a distant phenomenon that has nothing to do with me.), contemplation (death is closer to me than I thought and I should prepare for it), preparation (I will talk to my mom more often because we never knew what could happen), action (I am driving more carefully now), and maintenance.

## Results

We performed eight games in total. The games lasted for 98-120 minutes. After-game focus group interviews lasted 19-35 minutes while semi-structured interviews took 23-38 minutes. All participants (48) are second year medical students from Walailak University School of Medicine. 26 (54.17%) of them were male and 22 (45.83%) were female with the average age of 20 years old. Twenty students (41.7%) recently lost someone close to them within one-year period. Six of them (30%) stated that their loved one’s death had changed their perspective of death ( Table 1).

**Table 1.** Participant characteristics (n = 8 games)

Participant Characteristics	n = 48
Gender	
Male n (%) Female n (%)	26 (54.2) 22 (45.8)
Age mean (SD)	20 (0.6)
Have lost someone close to them within one year	
Yes n (%) No n (%)	20 (41.7) 28 (58.3)
Their loved one’ death changes their perspectiveof their death	
Yes n (%) No n (%)	6 (30) 14 (70)

As for the students’ bahavioural change using TTM model assessment, before the card game session, 42 (87.5%) of the students were in pre-contemplation stage while six (12.5%) of them were in their contemplation stage. After the game, 15 (31.25%) of the students changed their behaviour to contemplation stage while 33 (68.75%) shifted to preparation stage. After three months, follow-up phone calls revealed that none of them reverted to precontemplation stage while 12 (35%) and 32 (66.7%) of them were in contemplation and preparation stage respectively. Four (8.3%) of them went into action stage ( Table 2).

**Table 2.** Students stages of change regarding preparation of death

TTM stages of change	Pregame n (%)	Postgame n (%)	Three months’ postgame behaviour n (%)
Precontemplation	42 (87.5)	0	0
Contemplation	6 (12.5)	15 (31.25)	12 (25)
Preparation	0	33 (68.75)	32 (66.7)
Action ^*^	0	-	4 (8.3)
Maintenance ^*^	0	-	-

TTM = transtheoretical model

^*^ Action and maintenance stages behaviour could not be assessed immediately or at three- months period after the game.

For the content analysis relating to the card game, five primary themes with eleven subthemes emerged ( Table 3).

**Table 3.** Primary themes and subthemes from the card game

	Themes	Subthemes
*Pregame attitude of death*	1) Death is a distant phenomenon	1.1) Magic of being young and healthy 1.2) Not my cup of tea talk
	2) Fear of death	2.1) Fear of the unknown 2.2) Unexpected nature of death
*Card game attitude*	3) Safe space to disclose personal issues	3.1) Friendly facilitators 3.2) Bonding with classmates
	4) Seeing the world through different views	4.1) Constructive views 4.2) Shared learning experiences
	5) Surprise elements	5.1) Surprised questions 5.2) Simulated situation
*Postgame attitude of death*	6) Death distant closure	6.1) Death is closer than I thought 6.2) Impact of your death to others
	7) Changed behaviour	7.1) Mindfulness and self-reflection 7.2) Getting more connected with loved ones 7.3) ACP

TTM = Transtheoretical model

ACP = Advance Care Plan

### Pregame attitude of death

*Theme1: Death is a distant phenomenon*

Before the game, majority of the students (42) did not think of their death at all. They viewed the concept of death as a vague and distant event that could not relate to them in anyway.

*Magic of being young and healthy*

They also expressed that it is due to their youth that make it further away from their thought.

*“I am still young and healthy. It [death] won’t come to me soon.”* Student 4

*Not my cup of tea talk*

Others (8) stated that talking or even thinking about it make them feel uncomfortable and rather avoiding the subject.

*“It just feels weird talking or thinking about it while we can talk about something else.”* Student 2/ Focus group 2

*Theme 2: Fear of death*

*Fear of the unknown*

Another thought that make students feel uncomfortable talking about death is that they never know what they would meet.

*“I fear it [death]. Because we never know what could happen after that. It could be heaven or hell up to your religious belief or nothing at all. I guess “not knowing” is what terrifies me, not the death itself.”* Student 6

*Unexpected nature of death*

12 of them also mentioned the fear of talking about death due to its uncertainty nature.

*“It could be any moment [death]. Tomorrow, next year, that’s why it is so terrifying.”* Student 4/Focus group 8

These findings coincide with students’ TTM that 87.5% of them were in pre-contemplation stage and had no intention of thinking nor preparing about ACP at all. However, after the game, their attitudes changed drastically.

### Card game attitude

*Theme 3: Safe space to disclose personal issues*

All students (48) praised the card game for making a safe space for them. They could express their inner thought freely and safely without fear of being judged thanks to the facilitators and their classmates.

*Friendly facilitators*

*“I feel safe to talk about anything during the game. Because she [facilitator] teaches me and we are very close.” “Me too. She is friendly and down-to-earth so we can talk it out without fear of being judged.”* Focus group 7

*Bonding with classmates*

“I feel like I know my friend better. She is always a cheerful girl but I never knew that she has been through a lot at home.” Student 2

*Theme 4: Seeing the world through different views*

*Constructive views*

35 students mentioned about how this session make them see certain issues differently such as death.

*“There can be several answers to a question. For example, when we talked about our place of death. I want to die at home but my friend did not want to. He said that those who were left behind would be devastated. And both of us aren’t wrong.”* Student 1/Focus group 2

*Shared learning experiences*

They also liked the shared learning element of the card game, especially experiences from facilitators.

*“Some questions we could only see them from only one angle” “A kid’s view.” “Yeah. And he [facilitator] opened our eyes.” “Right? Like when we talked about our last meal. I never knew that dying people could not eat much.”* Focus group 3

*Theme 5: Surprise elements*

Apart from that, they were also fond of the surprise elements in the game. It helped to stimulate situations they had never thought of before.

*Surprised questions*

*“Most questions are what I had never thought of before. Like, what would you do if you were to die tomorrow? I never thought of that and there are lots of things to prepare.”* Student 7/Focus group 1

*Simulated situation*

*“I prefer this card game over regular lectures. I can be totally into it; exchanging ideas with friends as to what would I do in certain situation unlike lectures class where we can only learn the principles.”* Student 2/ Focus group 6

### Postgame attitude of death

*Theme 6: Death distant closure*

48 of them viewed death as closer to them than they had thought, reflecting the change in TTM into contemplation and preparation stages in all students.

*Death is closer than I thought*

“I can see now that death is closer to us than I thought. I thought that we will die at 80s or 90s but after this game, I know that that is not always the case. You could pass away just mere moments later. *Poof*. Just like that.” Student 6

*Impact of your death to others*

They also realised that one’s death brought larger impact to people, especially their family.

*“Death involves so many people. For example, if I die, my parents would be heartbroken. And who’s going to take care of my cats too? My parents are one thing but my cats depend on me solely.”* Student 5/ Focus group 2

*Theme 7: Changed behaviour*

While only 15 of them changed into preparation stage of change after the game, 33 of them changed to contemplation stage, rethought about death, and also considering changing their behaviour.

*Mindfulness and self-reflection*

Though they have not formed any concrete plan yet, 15 students considered the need to prepare for one’s death.

*“I feel like I need to prepare for it [death]. Like I need to do more about it.” “Yeah, me too. ‘cos we never know.”* Focus group 1

*“I always drive very fast, passing through the red light and stuff but I will drive slowly and more carefully now. Because we never know what could happened.”* Students 3/ Focus group 1

*Getting more connected with loved ones*

Majority of the students (31) also expressed the need to be more connected to their loved ones.

*“[last wish card] I will be a better daughter and talk with my grandma more often.”*

*Advance Care Planning (ACP)*

4 students stated their plan to make ACP with their family afterward.

*“[last wish card] I will make a will, save money for my cats, and ask my parents about questions in the cards.”*

## Discussion

ACP is one of the key elements of comprehensive palliative care. However, previous studies showed lack of knowledge and confidence to initiate one among doctors ( Bowden *et al.*, 2013) and medical students ( Kelly and Nisker, 2010; Suvarnabhumi *et al.*, 2013). Several suggestions have been made to improve palliative care core competencies in the undergraduate curriculum ( Anderson *et al.*, 2008; Kelly and Nisker, 2010; Wechter *et al.*, 2015) especially in communication skills and the Life Unlocking Card Game is a pilot study following those suggestions. The results showed that not only the card game made the students more aware of the concept of death after they played the game, but it also made them change their behaviour as well.

Following theme 1 and 2, participants reflected that before the game they saw death as a far-away phenomenon or found it too terrifying to talk or think about. However, the game changed their perspective drastically. Not only students being able to see death as a different view, that it could happen to anyone, it also leads to changes in their behaviour after the game at the three-months period. They also realised the importance of ACP, leading to initiating of ACP in 4 students, and the shift of TTM from pre-contemplation stage to contemplation, preparation and even action stage of change in 4 students afterwards. Themes 3 to 5 showed that students viewed the card game as a tool to create a safe space to talk within the group. It also helped them learn to see certain issues during the game such as death through different viewpoints, coinciding with constructivism theory of learning ( Dennick, 2016). Findings from this study also showed that this teaching method helped preclinical year students, who have no actual experiences with patients; it exposed them to complex issues during the end of life care such as medical decision making or ACP in a safe teaching environment.

Regarding this teaching method, all (48) students reflected on theme 3 that the card game created an engaging and safe environment for a serious topic such as death and dying. It also made them see the different perspective from other players, as shown in theme 4. Apart from that, the majority (45) of them preferred this teaching method over the conventional, didactic lecture. Results from this study showed that the Life Unlocking Card game is an effective tool to initiate an active learning method for serious topics such as death and dying due to its safe environment for participants and surprising elements. Still, this pilot study only demonstrates second-year medical students’ attitude of death and its effectiveness to teach about death and dying. Further study is needed to determine whether students, who have early exposure to end of life experiences, have a better understanding of end-of-life care or not ( Anderson *et al.*, 2008; Wechter *et al.*, 2015).

To our knowledge, this is the first study to conduct this learning method in preclinical year students in Thailand. The results show that not only the card game help students to have a better understanding of death and dying issues, it also changes their behaviour into contemplation, preparation, and action stage of change regarding their ACP at 3 months period after the session. We also use multiple methods to increase the rigour of our work; investigator and methodological with data triangulation methods.

Still, our work has several limitations. Since this is a pilot study to stimulate an active learning environment, we used convenient sampling method to include all second-year medical students. Thus, we have no control group to compare the result of the traditional teaching method such as didactic lectures to compare the effectiveness of each method. Furthermore, this session was performed with pre-clinical year students, all of whom had no experience with dying patients. Results may differ from clinical-year students who have an opportunity to observe or be involved in caring actual patients. Furthermore, we need large numbers of facilitators to engage with students (8 facilitators in total) because they are of the same age group so their views of death are not much differed. Hence, the need for facilitators to give them a broader perspective from adults who have been through losses.

## Conclusions

The Life Unlocking Card Game proves to be a useful tool to teach second-year medical students in death and dying and ACP issues. We found that students have a better understanding of the concept of death, changed their behaviour, and have positive experiences during the game. Further study in clinical year students or postgraduate students is recommended.

## Take Home Messages

• It is possible to teach sensitive issues such as death and dying in pre-clinical year medical students.
• Key elements are using a safe environment, a proper-tool, and a good facilitation.

## Notes On Contributors

Dr Tharin Phenwan is a Family Medicine lecturer at Walailak University School of Medicine. His research focuses on palliative care, qualitative research, interprofessional, and medical students assessment.Tanida Apichanakulchai is one of the leading members of Buddhika Foundation, a non-profit organisation that aims to raise death awareness and also the concept of peaceful death. Her work focuses on social engagement, volunteerisms, and mindfulness.Ekkapop Sittiwantana is also one of the members of Buddhika Foundation, a non-profit organisation that aims to raise death awareness and also the concept of peaceful death. His works focus on building social awareness.

## Declarations

TP is a lecturer at the school of medicine and teaches the medical students there.

## Appendices

### Appendix I. Life Unlocking Card Game Question List

**Table:**

Category	Questions
**Warm-up questions**	1. What are your worries about playing this game? 2. What is your expectation from this game?
**Emotional respite**	- What kind of activity do you spend most time on lately? - How much money would you want in your entire life? Why? - What or who is your source of strength? - What or who is your emotional support? - Define the first three words when you heard the word “palliative care” - What do you think happens after death? - Which event always makes you feel pleasant and happy whenever you think of it? - Are there any past events in your life that you wish to do differently? - Design your ideal funeral. - How long will you live? Why do you think that? - Who will you give the usernames/passwords of your online social accounts to (FB, IG, Twitter, etc) when you die? Why? - What is your dream that could **never** come true? - If you were terminally ill, which celebrities would you want to visit you? - What kind of last meal would you want? - Who would find it very hard to accept and let go if you were dead? - What are the most important things that you want to achieve no matter what before you die?
**Death and Dying issues**	- Define your “good death?” - Think of someone you haven’t met for more than one year, who do you want to meet before you die? - Have you ever witnessed a life-prolonging treatment such as CPR, intubation? Can you describe your experience? - Who’s dependent on you right now (financially, psychologically) and may have difficulties if you die? - If you were to die tomorrow, how would you manage your financial assets? - If today were your last day, who is the first person you would apologise to? And who is the first person you would express your gratitude to? - What kind of items would you give in your funeral? - If you had 6 months to live, who will you tell the news? - What is your strength to help you achieving a good death? (working as a palliative care provider, already make a living will, etc) - If you were lost in the sea, who will make the decision as to how long and how much money your family would pay to search for you? - If you could manage one thing on your last day, what would that be? - Is your home a good place to die? Why? - Think about someone you love who already passed away, what are your memories of them? - If you were diagnosed with a terminal illness, who would you turn to for advice? - How much money would you spend on your funeral? - How old were you when you witnessed your first death? How was your experience? - When did you realise that eventually, you, too, will die? - What is the most important thing you want to give to others when you die? What is that? Whom will you give that too? - Where do you want to die? Could you describe that? - If you were to sleep tonight and never wake up again, how prepared are you? What makes you feel that way?
**Medical decision making**	- If you were incapacitated and could not go to a toilet by yourself, who do you think will help you with your daily activities? And who do you think will **never** ask for their help? Why? - If you were terminally ill, e.g. could not communicate and were bedbound, who do you want to be your proxy regarding your treatment? - Who do you want to be with you when you stop breathing? Is there anything you want to say to them? - Will you donate your body as a cadaver? Why or why not? - After you die, how would you want your body prepared? - If you were terminally ill and your heart stopped beating, who would be your proxy regarding your treatment? **Is he or she aware of that?** - If you were terminally ill and needed a CPR, do you want the health care team to do that? Why? - If you were terminally ill, what kind of care do you want? (comfort only, symptoms free, etc.) - If you know that your loved ones have a terminal illness and may die soon, would you tell them? Why or why not?
