# Supplementary material for: Life unlocking card game in death and dying classroom for medical students
Source: MedEdPublish (2016). 2018 Aug 22;7:181. [Version 1] doi: 10.15694/mep.2018.0000181.1 (PMC10701803; doi:10.15694/mep.2018.0000181.1)
Supplement: Peer review history

# Reviewer report: Reviewer response for version 1

**DOI:** 10.21956/mep.19638.r29260

**Published:** 2018-08-24

**Authors:** Jayne Garner

**Affiliations:** 1 university of liverpool

This review has been migrated. The reviewer awarded 3 stars out of 5 I think the paper highlights the use of ‘gamification’ to explore student attitudes and experiences relating to death in a non-threatening and accessible way. Some of the focus group themes around uncertainty and the appreciation of no right and wrong personal attitudes to death are really insightful. The use of similarly styled ‘games’ as learning and discussion tools can be transferable regarding topic and perhaps to promote interdisciplinary discussion. I found this paper really useful and accessible, and the authors appropriately acknowledge the limitations of their sampling/population. I am definitely interested in exploring these ideas in future curriculum development.

Reviewer Expertise:

NA

No decision status is available

# Reviewer report: Reviewer response for version 1

**DOI:** 10.21956/mep.19638.r29259

**Published:** 2018-08-24

**Authors:** John Cookson

**Affiliations:** 1 University of Worcester

This review has been migrated. The reviewer awarded 4 stars out of 5 I also enjoyed this. I presume that the cards produce a randomness to the process which enables people to talk more openly. It would be interesting to know if those who had been through this process found it easier to talk to patients about death when they were further on in the course.

Reviewer Expertise:

NA

No decision status is available

# Reviewer report: Reviewer response for version 1

**DOI:** 10.21956/mep.19638.r29258

**Published:** 2018-08-23

**Authors:** Trevor Gibbs

**Affiliations:** 1 AMEE

This review has been migrated. The reviewer awarded 3 stars out of 5 A really interesting paper to read that uses a relatively simple card game to bring about active learning in the areas of personal values / beliefs about death and dying. I though this was pitched at an appropriate level for relatively new students, but I can easily see its potential for the more advanced clinical students. I feel that the paper can be improved by:- giving an example of the writings / questions on the cards- perhaps exploring how the students might perceive the effect of this new learning on future patients ( bearing in mind that they are pre-clinical) The authors have rightly explored the limitations of this paper but should be encouraged to explore this research further- on a greater number of students and taking it through to a clinical perspectiveA paper of interest to anyone involved with curriculum planning.

Reviewer Expertise:

NA

No decision status is available
